# Remarks on the Gibraltar Epidemic

**Published:** 1831-08

**Authors:** D. Barry


### *Remarks on the Gibraltar Epidemic.* By D. Barry, M.D.

(Continued from page 23.)

21st August. The boy Cafiero, the companion of the Fani children, was this day admitted into the Civil Hospital, as febris remittens, as already mentioned in my paper read before the College of Physicians.

23d. Mr. Fraser's own maid, Maria, taken ill whilst washing in the hospital washhouse.* This event was fixed upon her sister's memory, who was employed in the hospital at the time, and whom I examined on the subject in 1829, because it marked a monthly epoch in the age of her own baby, as I have already stated in a former number.†

29th. Under this date, the ominous words "yellow fever" appear for the first time in the annals of the late epidemic, thus:

"To H. Fraser, Esq., Civil Hospital.

"Sir: I beg to inform you, that Mary Silcock, of the Protestant persuasion, has a severe attack of yellow fever, and requires instantly to be admitted into hospital. (Signed)

"G. Woods, Hospital Assistant."

* In the Morning State 1st September, this woman is returned "ill more than a week;" in that of the 3d, "remaining;" on the 4th, "well." See the Morning States of these dates.

† Before the abettors of the local origin of the late Gibraltar fever can persuade the public that one or more cases of the disease occurred, in 1828, previously to the admission of the Dygden, they must come to a better understanding with each other, than they appear to have at present: they must bring forward some proofs of that fact, more convincing than their own bare, uncourteous assertions; and, above all, they must not overlook their own reiterated written acknowledgments to the contrary. Here are a few illustrations.

1. M. Chervin, up to the publication of his letter to M. Montfalcon, dated 1st June, 1829, could not discover that any case of the "maladie suspecte" had occurred before the middle of August. In 1830, after Mr. Fraser had published his very dignified observations on my paper read at the College of Physicians, M. Chervin roundly asserts, in a note appended to his translation of Mr. Wilson's Historical Sketch, p. 42, that the date assigned by Mr. Wilson, viz. "towards the close of August," to the attack of Mr. Fraser's maid, mentioned by him, actually refers to *a relapse;* and he gives the following words in English, as if from his author: "*she suffered a relapse on the 27th August.*" "Mais sa première attaque eut lieu le 4 ou le 3 du même mois." There is not a syllable of this in Mr. Wilson's original, which I have most carefully examined; and, indeed, Mr. W. himself denies all knowledge of the facts here asserted, in his letter quoted by Mr. Fraser. Nor can M. Chervin have had the information from Mr. Fraser, as he states, "à la fin de l'épidemie;" because, if he had, he would have written differently to M. Montfalcon in 1829.

2. Mr. Fraser, at his examination before the Board of Commissioners, stated

This note, transmitted by Mr. Fraser to John Duffield, Esq., one of the governors of the Civil Hospital, was carried by the latter, with just alarm, and without loss of time, to his Excellency Sir George Don, who seeing the terrible announcement, under the hand of a British medical officer, that this awful scourge was actually within the walls, wrote at the foot of Silcock's admission ticket, dated as above:

"Let the woman be admitted without delay, and placed in a separate ward. (Signed) "Geo. Don,
"General and Governor, Civil Hospital."

Thus clearly indicating the kind of sanitary measures he would willingly have adopted: but Mr. Duffield, on his way to the lieutenant governor, had met Dr. Hennen, and shewn him Mr. Woods' note; and Mr. Woods himself had written to Dr. Hennen the following:

"29*th August*, 1828.

Sir: I beg to report to you, that another case of *bilious remittent*, or yellow fever,* has shewn itself in my district.
(Signed) "G. Woods, Hosp. Assist."

that he could bring forward forty or fifty sporadic cases of yellow fever, from the sanitary annals of Gibraltar, and from his own practice: but, when pressed to specify these cases, he sent in a list of twenty-five, made up from 1824 to the end of 1826, purporting to have been "treated" in the Civil Hospital, of which he had been surgeon during that time. The letter in which this list was enclosed is dated 29th February, 1829.

At the foot of the list, or return, in Mr. F.'s own handwriting, and signed by himself, are the following words, viz.

"During the year 1827, there was not a case that came under the head of yellow fever: in the year 1828, *two cases*, bearing a strong analogy to the disease, presented themselves. (Signed) "Hugh Fraser."

3. In Mr. Fraser's general (nominal) return of patients affected with the epidemic fever, admitted, discharged, and died at the Civil Hospital, during the year 1828, the first name and earliest date are "Juan Cafire, (Cafiero,) admitted 21st August." This return is signed, and carefully corrected, by Mr. F.'s own hand.

Of this boy's case, (Cafiero,) Mr. Wilson, second surgeon of the Civil Hospital, writes thus, in his Historical Sketch, [see the Lancet for June 5th, 1830, p. 381,] "But it is a fact that a case of fever was admitted into the catholic division of this institution, so early as the 21st of August, and he is stated to have been one of those who visited a ship in the bay on the 9th or 10th of the same month. Taking it, however, as the first case of fever which appeared in the hospital, and there is no doubt that it was such."

Let these extracts be compared with Mr. F.'s official reply to the Board of Commissioners as to his first case, already published in your Journal, and also with his account, in your April number, of the *four cases of yellow fever* treated by him in the Civil Hospital in 1828, before the admission of Cafiero, or the attack of the Fani family!

* In the book kept open at Dr. Hennen's office, entitled "Medical Officers' District Reports," the following is written, in Mr. Woods' handwriting:

"29th August. Mary Silcocks, ætat. thirty; febris remittens; sent to hospital. (Signed) "G. Woods."

It is somewhat curious that Dr. Hennen should also use the words "yellow fever," for the very first time, in a letter of this date, to his Excellency the Lieutenant-governor, published by Mr. Fraser: but that very letter, by mingling together the more alarming with the harmless nosographical titles, after the manner of Mr. Wood's note to Dr. H., seems to have lulled the suspicions excited by the unqualified application of the term, in Mr. W.'s communication to Mr. Fraser. The only sanitary measures that followed, (and it is remarkable that precautionary measures are mentioned for the first time this day,) were directed solely to the drains and privies, at the suggestion of Mr. Wilson.

Mrs. Silcock was a washerwoman, who associated with and washed for sailors: she died on the 1st September.* Her case is referred to as drawn up by Mr. Fraser at the bedside, in my paper published in your February number of last year. She appears, from Mr. Woods' official report to Dr. Hennen, to have been attacked about the 23d of August. The appearance of her liver after death is quoted by Mr. Fraser, as particularly characteristic of the Gibraltar fever.

September 1st. Mrs. Flinn, a nurse employed in the Civil Hospital, appears on the books admitted under this date, as "febris remittens." She it was who nursed the woman Silcock, though she at first refused to go near her, having been assured by Mr. Fraser's own maid, Maria, then convalescent, that she had the same disease upon her, from which she (Maria) had but just recovered: Mrs. Flinn, however, was persuaded by Mr. Wilson, and finally nursed Mrs. Silcock up to her death.† Mrs. Flinn is returned discharged on the 10th September; had another attack at a later period of the epidemic, and died on the 13th October. The following is an abridgment of both her cases, as drawn up by Mr. Fraser himself at the time, and entered in his own handwriting, in the Civil Hospital casebook.

"1st September, 1828. Mrs. Flinn, Protestant nurse, attended Mrs. Silcock: taken ill today with severe headach and vomiting;

* Garcia's mother in law, (to be mentioned hereafter,) who died in September, and who washed the corpse of one of the Fani children, gave the following particulars to Mrs. Gonzalez, who stated them to the Anglo-French commission, on the 29th January, 1829.

"The mother of the deceased children (the Fanis) had in her house some foul linen to be washed, which Garcia's mother in law saw, and which had been brought by some Americans: but the children being taken ill, the linen was sent to an English woman on the road to Flat Bastion, who is herself dead."—D. B.

See Morning State.

† See Historical Sketch, Lancet.

is obviously very ill, but most decidedly much under the influence of fear. Bled to syncope. Still very ill; headach; tenderness of epigastrium.

"2d Sept. Irritability of stomach continues; rejects the effervescing mixture; cornea dull; *conjunctiva takes on a yellow tinge;* tongue moist, mucous coating in the centre; headach and epigastric pains continue; had a severe rigor at one o'clock, succeeded by clammy sweat.—3d. Headach trifling.

"4th. Much better.—6th. Decidedly convalescent."

*Mrs. Flinn's Second Case.*

"2d October, meridie. Mrs. Flinn, ætat. —. *This is a second attack.* Taken ill at eleven suddenly, with headach and præcordial pains."

This case is in the handwriting of Mr. Fraser, up to the 4th October; the remainder of the case is in Mr. Wilson's handwriting.

"6th October. Has passed a tolerable night, but is quite hysterical, from a constant nursing of apprehension.

"8th Oct. vespere. Is making herself worse with apprehension.

"10th, vespere. Tongue quite clean, moist, and of a perfectly healthy colour; still some hiccup; seems to rally. Has expressed a wish for some champagne.

"11th. No report.

"12th. Moribund.—13th. Expired this day."

"A correct copy, (Signed) "J. Millar,
"Assist. Staff Surgeon."

The above is the woman relatively to whom it is suggested, in Mr. Wilson's Historical Sketch, "that the resemblance of her symptoms, in her first attack, to those of Mrs. Silcock, whom she had nursed, resulted from the impression of fear which attendance on that case had made on her."*

29th August. A boy named Flinn, son of Mrs. Flinn, (not the nurse,) in whose house Mrs. Silcock had been ill for six days, was attacked this day: his brother was seized shortly after.—*Anglo-French commission.*

1st September. Manuel Garcia's child.† According to the testimony of the woman Villalonga, and her daughter, Maria Palheser, who lived in the room next to Garcia, whose room was divided from the apartment of the Fani family only by a thin board partition and a closed door, all opening into the same verandah, and by the admission of Garcia himself, the child was ill at this time. On this day the

* See Lancet, 5th June, 1830, page 382.

† Not noticed by Dr. Hennen.

Garcia family, consisting of one person who had passed the fever, and two women and a child who had not passed it, removed to the premises of Antonio Danino, Esq., where the child died on the 4th. The Garcia family, on coming to their new apartment, denied that they had resided in Fani's house, but confessed the fact afterwards. Both the women sickened about the 20th, and died, one on the 23d, the other on the 24th September,* as appears by a list of thirty-two persons attacked, and eighteen dead, in Mr. Danino's buildings, before and immediately after he had sent his people into camp. The first person attacked after the child's death in Danino's buildings, was on the 16th September. It may not be unimportant to the history of this epidemic, and it is justly due to the liberality of Mr. Danino, to state, that he spontaneously offered, and furnished gratuitously, the whole of the vehicles and drivers from his vast establishment, for the transport of the suspected population and their effects to the Neutral Ground, on the 5th September.

2d September. Maria Palheser's daughter, aged seven, sickened about this time:* she was in the constant habit of playing with the Fani children in the verandah. The grandmother had passed the fever before, and was not attacked. The three were sent to the camp on the Neutral Ground on the 5th, and did not return to town. Exactly twelve days after their removal, the mother, Maria Palheser, fell sick, and became yellow, as stated by her to the Anglo-French commission.

I shall now lay before you a chain of contagion, which, with that already given, will be found to connect all the cases as yet proved, by authentic records, to have occurred up to the turning out of the people on the 5th September: its elements are derived from facts noted on the spot, by persons who cannot be suspected of any bias, because the disease had not yet been recognised, nor was the approach of an epidemic visitation anticipated, as shall be fully proved hereafter.

Let it be borne in mind, that around this chain all was health within the territory up to the disturbance of the population on the 5th September, and that not even one case has been authenticated, which will not be found to constitute a link in its catenation.

Early in August, (according to Mr. Wilson's Historical

* Not noticed by Dr. Hennen.

Sketch, about the 11th,) some young persons were seized with fever, in the house of a Jew named Serfati.* This man's business consisted, as stated by A. Danino, Esq. to the Anglo-French commission, in trafficking with the crews of ships recently arrived in the bay. One of the children of a man named Acres, messenger attached to the civil secretary's office, who lodged with his family in Serfati's house, was attacked on the 15th August. The house of Mr. Martin, chief clerk in the same office, is distant from Serfati's "perhaps a hundred yards."† Mr. Martin being absent in England at that time, Mrs. M. was in the habit of sending frequent messages to Acres, who lived at Serfati's, and these messages were sometimes carried by one of her twin daughters. These two children were attacked, one on the night of the 17th, the other on the 18th August, and are mentioned by Mr. Fraser, in his official letter to the Board of Commissioners, dated 30th January, 1829, as the very first cases of the disease that had come to his knowledge in 1828.‡ In the course of the week, three or four more of the children were similarly attacked; and on the 23d August, Maria Moreno, the servant maid.§ Mrs. Martin, finding this woman very ill, and her disease being considered by the medical attendant, Mr. Fraser, as more severe than that of the children, though of the same description, it was resolved to have her sent to the Civil Hospital; but this the woman herself peremptorily declined, and was therefore removed, on the 25th August, to the house of a baker belonging to the commissariat, a Frenchman, named Androbet, where she died on the 28th August.|| This is the case mentioned by Mr. Wilson, in his official reply to the circular of the Board, just quoted, as "the first case I saw of the disease in question."

Mr. Serfati stated, that he believed that the Martin children used to play with those of Acres.

Mrs. Smith, wife of the master whitesmith of the ordnance

* It would have been better had Mr. Wilson given his authority for the date of these cases, as he was absent from Gibraltar at the time they occurred. —D. B.

† See Historical Sketch, Lancet, June 1830.

‡ See Mr. F.'s reply to circular, so often quoted.

§ See Lancet, May 29, 1830, p. 327; also Morning State, 1st September. —Dr. Hennen's letter, dated 29th August, published by Mr. Fraser, Medico-Chir. Review, September 1830: "And it is not a little remarkable, that the fatal case seen by me occurred in a woman who was a servant to Mr. Martin. (Signed) J. Hennen."—D. B.

|| See Mrs. Martin's deposition to the Anglo-French commission: see also the official Morning State of 1st September.

yard, lived with her family close to Androbet's house: she went, from charitable motives, to see Maria Moreno, once or twice during her last illness, and was attacked herself on the 29th August.*

Mr. Smith, her husband, sickened on the 2d September, and was the very first of all the people belonging to the ordnance yard who caught the disease: he continued to go to his work for the first day or two of his illness.

Mrs. Smith's daughter, nine years of age, was attacked about the same time: she slept with her mother.

The following is an extract from the evidence given by Mr. Smith before the Board of Commissioners, at their twenty-ninth sitting.

*Smith.* "I am acting master whitesmith in the ordnance department, and am generally employed in the grand store near Southport, in which were employed about twelve artificers, and as many labourers; of whom were taken ill, about six artificers and five labourers. I was the first taken ill myself. I lived in district 24; and my wife took ill before me, about three or four days."

The ordnance yard is situated at a considerable distance from the miasmal district, as it was sometimes quaintly called.

Androbet the baker, already mentioned, in whose apartment Maria Moreno died on the 28th, was seized with the disease himself on the 31st August, and was received into the Civil Hospital on the 2d September.† The following is an abstract of his case, copied from the books of that institution, where it is recorded in the handwriting of Mr. Peter Wilson.

"Androbet, ætat. fifty; 'febris remittens;' admitted 2d Sept. 1828, from the same yard‡ in district 24, where Mr. Martin's servant maid died: was seized with rigors, &c. on Sunday afternoon, 31st August, followed by vomiting: tongue loaded and tremulous; pulse eighty; severe pain of head; little thirst.

"3d. vespere. All pains of head and stomach gone.

"4th. three o'clock P.M. Makes no complaint, but the pulse has sunk almost into indistinctness.—Eight o'clock P.M. His pulse has now rallied, and is again distinct and regular.

"5th. A good night: tongue perfectly clean and moist; pulse regular."

To the complete copy of this case now before me, the following note is appended at this place.

"The foregoing is a true copy from the handwriting of Mr.

* See official Morning State for 3d September.

† See Morning State, 3d September.

‡ Mr. Wilson should have written "*same apartment*," and not "same yard."'—D. B.

Wilson, *who, it would appear, became indisposed at this period.** The remaining part of the case is in the handwriting of Mr. Fraser. (Signed) "J. Millar."

"8th September. Doing well.—11th. Is becoming *jaundiced*, —19th. Dismissed."

"A correct copy from the case-books, Civil Hospital."
(Signed) "John Millar, Assist. Staff Surgeon."

To the above I shall merely add, that Androbet's house, and all those around it in every direction, were perfectly healthy, except that of Teste, the health-guard, which was at a considerable distance, up to the arrival of the sick woman Maria Moreno, on the 25th August.

5th September. "Miss Francis died this day with black vomit."† The children of this and Mr. Martin's families were much together: they went to the same school. Miss F. visited one of Mr. Martin's daughters, about her own age, and sat with her frequently, during her (Miss Martin's) illness, before she was seized herself. Mr. Francis, the father of this girl, states, that he perceived a peculiarly oppressive and unusual smell about her person, which extended all over the house, during the last two or three days of her illness.‡ He is quite certain that there were no smells from drains, privies, nor from stagnant water: indeed, his house is remarkably clean, airy, commodious, and beautifully situated in a small garden.§

A little girl, aged thirteen, servant to Mrs. Winlo, who used to be sent to Mr. Martin's, to inquire for the children, during their illness, was attacked with fever about the end of August. Mrs. Winlo is now seventy-six years of age, (in 1829,) and has resided in her present house, which is large and commodious, since the year 1789. She and her family were intimate with the Martin's,|| and have always enjoyed good health.

* (Copy, Extract.) "*Civil Hospital; 6th September*, 1828.

"My dear sir: I went to bed last night, any thing but well, and during the night I have had two or three febrile touches, and this morning feel quite unable to stand; yet I do not feel very unwell.

"To Dr. Hennen." (Signed) "P. Wilson."

Mr. Wilson, in his Historical Sketch, acknowledges his conviction that one attack of the Gibraltar fever affords about the same protection from a future attack of the same disease, that vaccine affords against smallpox.—D. B.

† See Dr. Hennen's Morning State, 6th September.

‡ Extract from Mr. Wilson's letter to Dr. Hennen, just quoted: "Three more of Francis' children were seized last evening;" viz. 5th September.

§ See the documents of the Anglo-French commission.

|| See idem.

1st September. " P. Berte, febris." These words appear in the out-patient book of the Civil Hospital under this date, in the handwriting of Mr. Wilson, with this note appended to the prescription: "House under Mr. Martin's."

3d September. Mary Galt's daughter, aged twelve, the playmate of Mr. Francis' children, was attacked this day: she had no doctor, and recovered.*

Another servant maid, whom Mrs. Martin hired from the south district of the rock, was put to sleep in the bed which Maria Moreno had occupied; was seized with the disease at the end of a week; was sent to the Civil Hospital, and recovered. Her symptoms were exactly similar to those of her predecessor, as stated by Mrs. Martin to the Anglo-French commission. Mr. and Mrs. Martin, with their eldest daughter, had passed the fever in former epidemics, and were the only individuals of their family, consisting of ten persons, who were not attacked in 1828.

"The vault of the drain belonging to Mr. Martin's house is built of flags, joined together with Roman cement, so as not to allow any exhalation to escape. Mrs. Martin has never perceived any bad smell in her house; but somebody having spoken to her about the drain near the apartment of her daughters, she conducted Dr. Fraser to the opening of that drain, and into the apartment of her children; and that physician could perceive there no offensive smell, but he thought it would be proper to close the drain."

(Signed) "Chervin, d.m.p., Louis,
"Trousseau, D. Barry."†

The following declaration appears to be of some importance, with reference to Mr. Wilson's statement, already noticed, viz. " that two individuals of a Jew family, residing in the house in which John Whitelock, (Mr. Duguid's coachman,) died, were attacked before the latter."

Naricia, wife of Xalom Benzeerim, a Moorish Jew, stated to me, in 1829, and it was afterwards confirmed by her husband, "that her son Abraham‡ fell sick eight days before Whitelock, who lived in the apartment under hers, in the same house, on a Tuesday: shivering, red face and eyes, severe headach, pain at the pit of the stomach, five days' fever. The Misses Serfati were intimate with Mrs. Benzeerim, and had been to visit her on the Sabbath before her son fell sick: they staid about two hours. On the next day,

* Not noticed by Dr. Hennen.

† Extracted from the official translation of the documents of the Anglo-French commission.—D. B.

‡ Not noticed by Dr. Hennen.

Sunday, one of these young ladies was attacked in the morning, the other in the evening. The brother of the Misses Serfati came to Mrs. Benzeerim's house, to fetch a Jew quack doctor, named Moses Bocasis, to attend his sisters. This boy (the brother,) fell sick on a Wednesday or Thursday: he had been to fetch Bocasis before Abraham was seized. Mrs. Benzeerim herself fell sick on the evening of the day on which her son Abraham was attacked:* her other son, two years of age, slept with her that night, and fell ill next morning.† Mrs. Benzeerim further declares, that Moses Bocasis came to her sitting room, and staid with her about an hour and a half, after having applied leeches to the Misses Serfati: her son Abraham fell ill next morning."

Moses Bocasis himself was received into the Civil Hospital on the 3d September: his case, drawn up by Mr. Fraser, is now before me; the following is an extract:

"3d September, ten o'clock A.M. Just admitted, and from the dwelling in which Mr. Duguid's servant died: taken ill three days ago, with rigor and headach.

"4th. Aspect dusky, eyes greatly tinged. Died this day."

"5th September, Friday. Edward Acres, ætat. twenty-two; febris remittens. Is from a house, or rather hovel, near the Naval Hospital, but worked at Moir's limekiln, [in the infected district,] until the alarm of fever began; but on Sunday he was in the district, on the evening of which day he fell ill."

"A true copy, from the handwriting of Mr. Wilson, in the case-book, Civil Hospital. (Signed)

"J. MILLAR, Assist. Staff Surgeon."

This was the brother of Acres the messenger, already mentioned, and also of the reputed wife of Holdroyd, of the 12th regiment, (the first military case,) with whom he lodged in the infected district, until he found himself ill, when he fled to his mother's shed in the south, and remained there concealed until he was discovered on the 5th. Acres returned to his mother's, from the hospital, very ill. The family named Martinez, Portugueze, living in that shed with old Mrs. Acres, was the first attacked in that quarter; and those who visited them in their illness, named Casella, the next. (See the evidence of Mrs. Acres before the Board of Commissioners, and also of the woman Casella: the documents of the Anglo-French commission, &c.)

The very first cases of all epidemics are by far the most important; for it is only at the beginning, and near the

* Not noticed by Dr. Hennen. † Not noticed.

source of these visitations, that the transition of disease from place to place, and from person to person, can be traced, or the simultaneous bursting forth of many cases at distant and unconnected points, can be usefully marked. The meanderings and the irrigations of the rivulet can be easily followed, until its waters are lost in the great river, or lake. Thus, from the family of the low trafficking Jew, Serfati,* the stream of pestilence, at first small, is distinctly seen flowing through, and poisoning, the Martin family, Androbet, the Smiths, the people of the Ordnance yard and their connexions, where it is lost: again, from the Martins to the families of Francis and Galt; from the family of the smuggler and bumboat man to the Civil Hospital, the Garcia, Palheser, and Danino families; from the house, the best in the district too, where the Benzeerims, the Whitelocks, the Leahys, Bocasis, and the Benheims, were successively attacked, to their next-door neighbours; from the dissolute washerwoman Silcock, to the young persons of the house in which she had been four days shut up with the disease upon her, and to her hospital nurse; from Acres, to the family inhabiting the wooden cottage to which he fled in the south, nearly two miles distant from the infected district.

But when the steaming emanations of a thousand sick of the same disease have so loaded the whole atmosphere of a confined and sheltered place like Gibraltar, rendering it a sort of pond or lake of infective effluvia, the track of the stream of pestilence is lost: then it is that many of the predisposed will be attacked, who may have had no personal intercourse with the sick, particularly after night exposure near the infected dwellings, when there is neither wind to disperse, nor heat sufficient to rarefy and raise the morbid emanations from the earth. Then it is that many of those, whose want of predisposition has enabled them to resist this aerial source of contagion, will also resist occasional intercourse. It is, therefore, a fallacious mode of reasoning against the communicability of the Gibraltar fever, to adduce examples either of attack independently of personal intercourse with the diseased, or of exemption from attack notwithstanding such intercourse; more especially if these

* "There is a ship in quarantine, with the plague on board. A smuggler's family is first attacked, or the family of a health guard, or washerwoman. There is a rumour that these people had communication, but there is no direct proof: the communication was penal, therefore secret; no one will come forward to prove it."—(Gooch, on the Question "Is the Plague a Contagious Disease?")—D. B.

examples be taken from the acmé of the epidemic: one positive is worth a thousand negative facts.

How has it happened that not even one of the medical officers and servants attached to the London Fever Hospital has escaped at least one attack of the disease during their attendance; yet that many visit that institution, and many permanently reside next door to it, yet have never been attacked? The frequency, the proximity, and abundance with which the morbid germs are applied to the mucous surface of the lungs, under all states of predisposition, will account for this most interesting fact. The following extract from a lecture on Contagion, by my learned and esteemed friend Dr. Tweedie, senior physician to that hospital, is most highly deserving of attention.*

"I can state, from incontrovertible evidence, that, with one exception, every physician who has been connected with this establishment has been attacked with fever, and that three out of eight have died since the establishment of the hospital in 1802; and since my appointment, five years ago, I have been twice attacked. The domestic establishment, too, have one and all passed through fever, including matrons, apothecaries, porters, domestic servants, and all the nurses; and, to show that the contagious principles may be engendered by fomites in clothing, the laundresses who wash the patient's linen are constantly attacked; so that it is with difficulty that women can be found to undertake this loathsome, and frequently disgusting duty.

"Now let us compare these facts with what occurs at the adjoining establishment, the Smallpox Hospital, which stands within a few feet of the Fever Hospital, the two buildings forming two sides of a square. I am informed by Dr. Gregory, the present attending physician, that, for the last seven years, fever is unknown in the establishment."

* See the Lancet for September 22, 1827, p. 780.

☞ Dr. Barry will conclude his Remarks on the Gibraltar Epidemic, as soon as possible after his return from Russia, where he has been sent on a mission, with other physicians, to investigate the nature, &c. of the cholera, or the malady that has been prevailing there.—Editor.

